# Role of adiponectin, resistin and monocyte chemo-attractant protein-1 in overweight/obese asthma phenotype in children

**DOI:** 10.1186/s12887-023-04046-6

**Published:** 2023-05-06

**Authors:** Abeer M. E. Osman, Ayat A. M. Motawie, Amany M. Abd Al-Aziz, Nadia A. A. Mostafa, Nehal S. Hasan, Mohamed S. El-Baz

**Affiliations:** 1 Pediatric Department, National Research Centre, Cairo, Egypt; 2 Pediatric Department, Faculty of Medicine, Cairo University, Cairo, Egypt; 3 Clinical and Chemical Pathology Department, National Research Centre Cairo, Cairo, Egypt

**Keywords:** Asthma phenotypes, Obesity, Adiponectin, Resistin, MCP-1

## Abstract

**Background:**

Asthma is a chronic inflammatory disorder of the airways with diverse overlapping pathologies and phenotypes contributing to a significant heterogeneity in clinical manifestations***.*** Obesity may modify asthma risk, phenotype, and prognosis***.*** A suggested mechanism linking obesity and asthma is through systemic inflammation. Adipokines secreted by adipose tissue were suggested to provide a link between obesity and asthma.

**Objective:**

To have an understanding for the contribution of adiponectin, resistin and MCP-1 to development of distinct asthma phenotype in overweight/obese children through assessment of their serum level and correlation to pulmonary function tests.

**Subjects and methods:**

The study included 29 normal weight asthmatics, 23 overweight/obese asthmatic children and 30 controls. All cases were subjected to detailed history taking, thorough examination and pulmonary function tests. Serum adiponectin, resistin, MCP-1 and IgE were assessed to all recruited subjects.

**Results:**

Adiponectin level was significantly higher in overweight/obese asthmatics (24900 ± 1600 ng/ml) compared to normal weight asthmatics (21700 ± 1700 ng/ml) and control (23000 ± 3200 ng/ml), (*p* < 0.001 & 0.051 respectively). Normal weight asthmatics had significantly lower adiponectin level than control, (*p* = 0.039). A significant low level of MCP-1 in overweight/obese asthmatics (149.5 (20—545) ng/L) compared to control (175 (28 -1123.5) ng/L), *p* = 0.037. No significant difference was found regarding resistin. Normal weight asthmatics had significantly lower FEV_1_% and FVC% compared to overweight/obese asthmatics (*p* = 0.036, 0.016 respectively). A significant positive correlation was found between (FEV1%, FVC) and BMI in normal weight asthmatics (*P* = 0.01, < 0.01 respectively) and a significant negative correlation between PEF and BMI (-0.42, *p* = 0.05) in obese/overweight asthmatics. Resistin/adiponectin ratio was not affected by sex, degree of asthma severity or level of asthma control in either normal weight or overweight/obese asthmatic.

**Conclusion:**

This work could suggest that adiponectin may play a role in overweight/obese asthma phenotype where it is possible to have a dual action (pro & anti- inflammatory). It seems that resistin had no role in asthma pathogenesis.

## Background

Asthma is a chronic inflammatory disorder of the airways associated with airway inflammation and reversible airflow obstruction [1]. It is considered a syndrome, with diverse overlapping pathologies and phenotypes contributing to a significant heterogeneity in clinical manifestations [2]. Accumulating evidence points to localized inflammation in adipose tissue, which, in turn, promotes systemic low-grade inflammation as a primary force contributing to the development of different pathologies [3]. Obesity may significantly modify asthma risk, phenotype, and prognosis [4]. The inflammation was described to be T-helper 2 (Th_2_) mediated allergic inflammation in normal weight asthmatics and non (Th_2_) cytokine-driven, in overweight/obese asthmatics [5].

Adipokines secreted by adipose tissue was proposed to provide a link between obesity and asthma [6-8]. Adiponectin and resistin modulate pro-inflammatory environment in obesity [9]. They may alter T-helper 1 (Th_1_)/T helper-2 (Th_2_) balance, immune tolerance, lung development, airway smooth muscle and airway responsiveness which are associated with asthma development [10].

Adiponectin plays an important role in glucose and lipid metabolism and has both inflammatory and anti-inflammatory responses [11]; Studies have shown that levels of circulating adiponectin are altered in overweight/obese than normal weight patients with asthma [12].

Resistin is postulated to trigger pro-inflammatory response in-vitro and in-vivo [13]; at the same time, these pro-inflammatory agents can regulate resistin gene expression [14]. Resistin up-regulates the expression of monocyte chemo-attractant protein (MCP)-1 which have been known to be related to allergic inflammation [15]. Also, resistin has been linked to Nuclear Factor-kB (NF-kB); transcription factor linked to expression of many pro-inflammatory genes [16].

So, in this work we aimed to have an understanding for the contribution of adiponectin, resistin and MCP-1 to a possible development of distinct asthma phenotype in overweight/obese children through assessment of their serum level in children with asthma with normal and increased BMI and studying their correlation to pulmonary function test’.

## Subjects and methods

This randomized case–control study was carried out in allergy clinic, New Children Hospital, Cairo University and the medical service unit, National Research Centre (NRC). The sample size was 52 patients aged 6–13 years old, with chronic persistent asthma according to GINA guidelines (2016) [17] and thirty (30) age and sex matched healthy non-asthmatic children with normal BMI as a control group. Written informed consent was taken from all patients’ guardians before enrolment in the study and after full explanation of their role in the research. The study was approved by the Medical Research Ethics Committee, National Research Centre (registration number: 12065) according to World Medical Association Declaration of Helsinki (2013) [18]. Data were documented in the patients’ files and on special excel sheets. Confidentiality on handling the database was guaranteed and privacy of participants was ensured.

Obese, overweight, or normal weight are defined according to body mass index (BMI) using Egyptian Growth Charts (2002) [19]. BMI is a person’s weight in kilograms divided by the square of height in meters (BMI = Weight (kg)/Height (m2) [20]. For children and teens, BMI is age- and sex-specific and is often referred to as BMI for age as follows [21]:

• Normal weight: BMI >5th - <85th percentile
• Overweight: BMI exceeded 85th - <95th percentile
• Obesity: BMI exceeded >95th percentile.

The asthmatic patients were classified into two groups according to their BMI.

• ***Group 1***: Normal weight asthmatics, included twenty-nine children with normal weight, BMI category (>5th - <85^th^ percentile).
• ***Group 2***: Overweight/obese asthmatics included ten overweight children (>85^th^ - <95^th^ percentile), and 13 obese children (> 95^th^ percentile).

Normal weight children aged 6–13 years, with BMI =  > 5^th^—< 85th percentile free from any disease were included as a control group.

### Inclusion criteria

Children aged from 6–13 years with persistant asthma of different degrees of severity (mild, moderate, or severe) based on *GINA* *criteria* (2016) [17]. Obesity was mainly dietary and not related to any endocrinal or chromosomal abnormality and was not associated with any morbidity.

### Exclusion criteria

Patient with any other chronic chest problem other than asthma or recent asthma exacerbation during sample collection or having other chronic illnesses including diabetes mellitus, kidney disease, liver disease or thyroid dysfunction or other endocrine diseases. Overweight/obese patients on diet program or suffering from obesity related morbidity.

All patients were subjected to detailed history taking ( *Personal history:* name, age, sex, residence; *History of asthma:* Onset, course and duration of symptoms, age of diagnosis, frequency severity & pattern of symptoms, other associating allergic conditions e.g. allergic rhinitis, precipitating and/or aggravating factors e.g. viral infections, irritants, environmental allergens, etc.; *Past history:* excacerbations, hospital admission, previous treatment; *Family History;* asthma, allergic condition; *Full clinical examination*: height, weight and body mass index, waist circumference, waist/hip ratio, mid upper arm circumference, medical examination: general and systemic (head & neck, chest, heart, abdomen, upper & lower limbs); *Pulmonary function testing* and *laboratory investigations* ( Complete blood picture, total serum IgE, serum resistin, serum adiponectin, serum monocyte chemo-attractant protein (MCP)-1).

#### Pulmonary function tests

Spirometric testing were done using Spirolab III spirometer and pulse oximeter (MIR company, USA) at National Research Centre. The results of spirometry were expressed as a percentage of the predicted value adjusted for age, gender, weight, height and race fulfilling the American Thoracic Society/European Respiratory Society (ATS/ERS) recommendations for spirometry (which are regularly updated).

##### Technique of pulmonary function test

The procedure was carefully explained to the patient, according to Johns and Pierce (2011) [22].

The test should be repeated until we obtain three acceptable tests that meet the repeatability criteria and the highest value was recorded, ideally with less than 0.15 L variability for forced expiratory volume in one second (FEV_1_) and forced vital capacity (FVC) between the highest and second highest result. To ensure an acceptable result, the FVC maneuver must be performed with maximum exhalation effort immediately following a maximum inspiration [22].

The Bronchodilator test (BDR) is assessed after 15 min of 4–5 doses of 100 μg salbutamol. A response to the bronchodilator is considered with > 12% improvement in FEV_1_ or 15–25% improvement in forced expiratory flow at middle half of FVC (FEF_25-75%_) suggesting reversibility of airway obstruction which is characteristic of asthma [23].

#### Interpretation of the spirometry results

 The results of spirometry were expressed as a percentage of the predicted value adjusted for age, gender, weight, height and race fulfilling ATS/ERS recommendations for spirometry. Our spirometer included age-specific predicted values.

Values of FVC (> 80% of predicted or above the lower limit of normal), FEV_1_ (> 80% of predicted or above the lower limit of normal), and FEV_1_/FVC ratio (FEV_1_/FVC) (> 0.90) are suggestive of normal spirometry [23, 24].

The obstructive pattern is usually characterized by decreased FEV_1_ (< 80% of predicted or below the lower limit of normal), decreased FEV_1_/FVC, FEF_25-75%_ below 60% of predicted and normal FVC (FVC may be decreased in severe obstruction) [23]. FEV_1_ increases by more than 12% and 200 mL (in children, > 12% of the predicted value) after inhaling a bronchodilator ‘bronchodilator reversibility’ that may be absent during severe exacerbations or viral infections [24]. FEV_1_ percentage is used in children to predict the severity of airway obstruction as follows: FEV_1_ > 80% indicates mild asthma; FEV_1_ 60 – 80% indicates moderate asthma; FEV_1_ < 60% indicates severe asthma [25].

### Laboratory tests: at National Research Centre

5 cc venous blood was collected from every case using standard venipuncture aseptic technique. 1 cc taken on EDTA tube for complete blood picture assessment and the rest was centrifuged and sera were collected and stored at -20^℃^ for assessment of serum adiponectin, serum resistin, serum MCP-1 & total serum IgE using Enzyme linked immuno-sorbent assay (ELISA) technique.

• Serum IgE (immunoglobulin E): enzyme immunoassay test kit provided by Bio Check, Inc.; Foster city, CA 94404, USA; for the quantitative determination of IgE concentration in human serum)
• Serum resistin: Enzyme-linked immunosorbent assay kit, for quantitative detection of human resistin, provided by Assaypro LLC 30 Triad South Drive St. Charles, MO 63304, USA)
• Serum adiponectin: ELISA kit, for quantitative sandwich enzyme immunoassay technique that measures adiponectin in less than 3 hours, provided by Assaypro LLC 30 Triad South Drive St. Charles, MO 63304, USA).
• Serum MCP-1: ELISA kit, for quantitative determination of human MCP-1 in human serum provided by glory science co., ltd. 2400 veterans Blvd. Suite 16 – 101, Del Rio, TX 78840, USA).

## Statistical method

Data management and analysis was performed using Statistical Package for Social Sciences (SPSS) vs. 23. Numerical data were summarized using means and standard deviations or medians and ranges, as appropriate. Categorical data were summarized as numbers and percentages. Numerical data were explored for normality using Kolmogrov-Smirnov test and Shapiro–Wilk test. Comparisons between two groups for normally distributed numeric variables were done using the student’s t-test while for non-normally distributed numeric variables were done by Mann–Whitney test. Comparisons between more than 2 more were performed by the one analysis of variance (ANOVA) for normally distributed variables and Kruskal–Wallis for non-numerical variables [26]. Chi-square or Fishe’s tests were used to compare between the groups with respect to categorical data, as appropriate [27]. To measure the strength of the association between numeric variables, the Spearman’s correlation coefficients were calculated [28]. To assess the diagnostic capability of the tests, the sensitivity, the specificity, and the area under the curve were calculated. The best cutoff value to differentiate between the groups was computed from the ROC curve. All *p*-values are two-sided. *P*-values < 0.05 was considered significant.

## Results

Descriptive data for included children (asthmatics and control) are summarized in Table 1.

**Table 1:** descriptive data of the studied groups: normal weight and overweight/obese asthmatics and control

**Variable**	**All asthmatics (52) percent%**		Controls (30) **percent%**	***P*-value**
	Normal weight (5th- < 85th percentile): (*n* = 29)	overweight/obese (*n* = 23)		
**Age (median- years)**	9.09 (6–13)	8.1(6–13)	9.0 (6–13)	0.66
Gender				
Male	33 (63.5%)		15 (50%)	
Female	19 (36.5%)		15 (50%)	
**Residence**				*P*-value between normal weight and overweight/obese asthmatics residency = 0.038
Urban 37 (71.2%)	82.8%	56.5%		
Rural 15 (28.8%)	17.2%	43.5%		
**BMI category**				
	29 (55.7%)	overweight:10 (19.2%)		
		obese:13 (25%)		
**Age at diagnosis (years)**	3.8 ± 2.9			
≤ 6 years	45 (86.5%)			
> 6 years	7 (13.5%)			
**Age of onset of obesity (years)**		4.5 ± 2.4		
**Severity of asthma**				
Mild	9 (31.0%)	5 (21.7%)		
Moderate	12 (41.4%)	14 (60.9%)		
Severe	8 (27.6%)	4 (17.4%)		0.375
**Level of asthma control**				
Well controlled	6 (20.7%)	3 (13.0%)		
Partly controlled	5 (17.2%)	9 (39.1%)		
Uncontrolled	18 (62.1%)	11 (47.8%)		0.204

No significant difference was noticed between normal weight and overweight/obese asthmatics concerning degree of asthma severity (*p* = 0.375) or level of asthma control (*p* = 0.204) or clinical presentation (wheezes, cough, difficult breathing & chest tightness) (*p* = 0.077) or possible trigger factor (e.g.exposure to air currents *p* = 0.143; animal fur, *p* = 1,000; exercise, *p* = 0.245). The use of oral steroids was significantly higher in overweight/obese asthmatics (56.5%) compared to normal weight asthmatic (17.2%), *p* = 0.003; this might be attributed to Egyptians parents and patients’ preference to receive oral rather than inhaled steroids.

The comparison of pulmonary function tests between asthmatic cases is presented in Table 2.

**Table 2:** Comparison of pulmonary function tests between normal weight and overweight/obese asthmatics groups

**Pulmonary function testing**	**Normal weight asthmatics**	**Overweight/obesity asthmatics**	
	**Mean** ± standard deviation	**Mean** ± standard deviation	***P*-value**
**Forced expiratory volume in first second (FEV**_**1**_**%)**	70.7 ± 20.2	84.7 ± 20.3	**0.036**
**Forced vital capacity (FVC %)**	72.0 ± 18.9	87.1 ± 18.3	**0.016**
**FEV**_**1**_**/FVC ratio** **(FEV**_**1**_**/FVC %)**	84.0 ± 18.1	84.8 ± 9.0	0.594
**Peak expiratory flow (PEF %)**	66.3 ± 21.4	78.2 ± 22.5	0.163
**Forced Expiratory Flow at middle half of FVC** **(FEF **_**25–75%**_**)**	69.7 ± 27.3	74.8 ± 25.8	0.665

Normal weight asthmatics had significantly worse FEV_1_% and FVC% compared to overweight/obese asthmatics, with *p*-value = 0.036, 0.016 respectively. However, no significant difference was detected for FEV_1_/FVC %, PEF%, or FEF _25–75_% between both groups.

The correlation between BMI and PFT among asthmatic groups are presented in Table 3.

**Table 3:** Correlation between BMI & pulmonary function test indices among normal weight and overweight/obese asthmatics

Pulmonary function testing	Calculated BMI			
	**Normal weight asthmatics**		**Overweight/Obese asthmatics**	
	r	*P*-value	r	*P*-value
**Forced expiratory volume in first second (FEV**_**1**_**%)**	**0.52**	**0.01**	0.27	0.23
**Forced vital capacity** **(FVC %)**	**0.63**	**< 0.01**	0.24	0.27
**FEV1/FVC ratio** **(FEV**_**1**_**/FVC %)**	**-0.09**	0.62	**-0.11**	0.60
**Peak expiratory flow** **(PEF %)**	0.13	0.50	**-0.42**	**0.05**
**Forced Expiratory Flow at middle half of FVC** **(FEF **_**25–75%**_**)**	0.16	0.39	0.19	0.39

A significant positive correlation between (FEV_1_%, FVC) and calculated BMI in normal weight asthmatic group, (*P* = 0.01, < 0.01 respectively); FEV_1_% and FVC improved (increased) when BMI increased. A significant negative correlation between peak expiratory flow (PEF) and BMI was detected in obese/overweight asthmatics. Yet, a negative correlation was found between calculated BMI and FEV_1_/FVC ratio in normal weight and overweight/obese asthmatics but statistically non-significant. The increase of BMI was associated with lower FEV_1_/FVC ratio (increased airflow limitation).

Comparison between the three studied groups (normal weight asthmatics, overweight/obese asthmatics & control) regarding laboratory investigations are summarized in Table 4.

**Table 4:** Comparison of laboratory investigations among normal weight, overweight /obese asthmatics, and control groups

	Normal weight asthmatics	Overweight /Obese asthmatics	Control	*P*-value
**Adiponectin ng/ml (mean + SD)**	21700 ± 1700	24900 ± 1600	23000 ± 3200	
				normal weight asthmatics vs control *p* = 0.039
				overweight/obese asthmatics vs control *p* = 0.051
				normal weight asthmatics vs overweight/obese asthmatics *p* < 0.001
**Resistin ng/ml (mean + SD)**	26.5 ± 9	29 ± 14.5	27.5 ± 11	control vs all asthmatics *p* = 0.758
				normal weight asthmatics vs overweight/obese asthmatics *p* = 0.747
**MCP-1 ng/L (median -range)**	149.5 (54—589.5)	149.5 (20—545)	175 (28 -1123.5)	
				normal weight asthmatics vs control *p* = 0.268
				overweight/obese asthmatics vs control *p* = 0.037
**IgE IU/ml (median- range)**	174.9 (22.9 -804.9)	140.4 (7.6 -831.5)	39.7 (0.1—153.6)	
				normal weight asthmatics vs control *p* < 0.001
				overweight/obese asthmatics vs control *p* < 0.001

Serum levels of adiponectin, MCP-1 and IgE revealed significant difference between the studied groups. Adiponectin level was significantly higher in overweight/obese asthmatics compared to both normal weight asthmatics and control. Normal weight asthmatic patients had significantly lower adiponectin level than control, also, a significant difference was found between normal weight and overweight/obese asthmatics. Although the median range for MCP-1 in normal weight and overweight/obese asthmatics was the same number; yet the range variation in each group led to a significant low level in overweight/obese asthmatics compared to control.

Conversely, overweight/obese asthmatics had higher resistin level than normal weight asthmatics and control, no significant difference was found.

Total serum IgE level was higher in normal weight asthmatics than overweight/obese group and both had significant higher IgE level compared to controls. Only 40% of the asthmatic groups had elevated eosinophilic count with no significant difference (*p* = 0.308).

Post hoc analyses showed a significant difference between normal weight asthmatics and Control (*p* = 0.039) and between overweight/obese asthmatics and Control (p = 0.051) for adiponectin. A significant difference was found only between overweight/obese asthmatics and control (*p* = 0.037) for MCP-1.

**Fig. 1:**
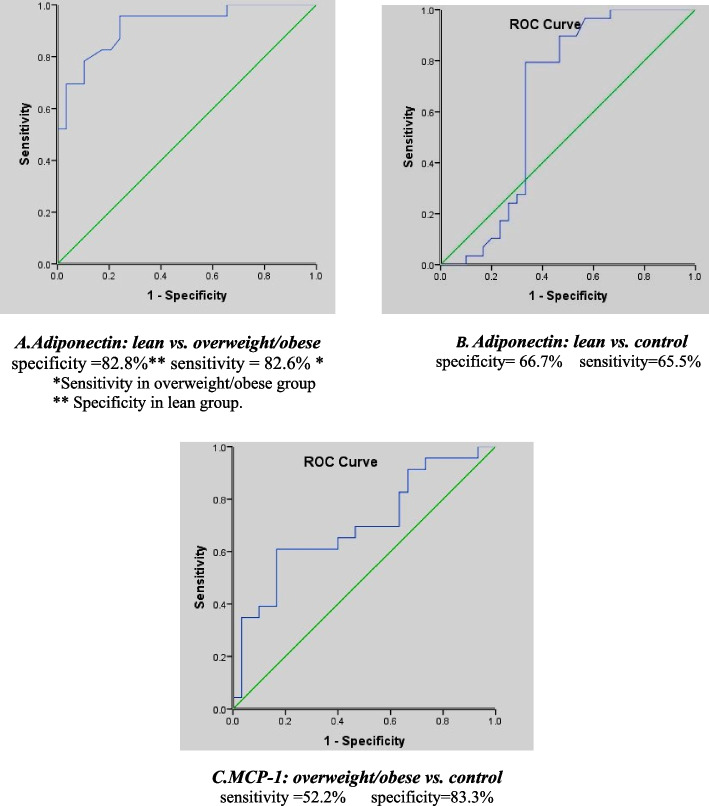
Roc curves for predictive value of adiponectin and MCP-1. **A** Adiponectin: lean vs. overweight/obese. **B** Adiponectin: lean vs. control. **C** MCP-1: overweight/obese vs. control

For the whole asthmatic groups, the correlation of BMI with pulmonary function tests showed a significant positive correlation between BMI and FEV_1_ (*r* = 0.474, *P* = 0.001), FVC (*r* = 0.552, *P* < 0.001). However, the correlation of BMI and laboratory investigations (MCP-1, adiponectin, resistin, IgE, eosinophils) showed a significant positive correlation only with adiponectin (*r* = 0.545, *P* < 0.001) and a weak negative correlation with eosinophils (*r* = -0.325, *P* = 0.034). To evaluate which adipokines can have a predictive diagnostic value, we performed Receiver Operating Characteristic analysis (ROC) as shown in Fig. 1.

The comparison between normal weight and overweight/obese asthmatics using ROC curve showed a cutoff value of 23525 ng/ml for adiponectin with 82.6% sensitivity in overweight/obese asthmatics (area under the curve = 0.920, *p* =  < 0.001) and a positive predictive value 79.2%. In normal weight asthmatics a negative predicted value was 85.7% and the comparison between normal weight asthmatics and control showed area under the curve = 0.658 with *p* = 0.037 (95% Confidence Interval: 0.506–0.811).

Resistin/adiponectin ratio was not affected by sex (normal weight asthmatics, *p* = 0.811; overweight/obese asthmatics, *p* = 0.308); degree of asthma severity (normal weight asthmatics, *p* = 0.455; overweight/obese asthmatics, *p* = 0.166) or level of asthma control (normal weight asthmatics, *p* = 0.213; overweight/obese asthmatics, *p* = 0.324).

## Discussion

No specific biomarker has been accepted to define obesity-related asthma; although adipokines have been proposed; perhaps because its role in obesity differs from TH_2_ and non-TH_2_ asthma [29]. The results of studies related to the effect of adiponectin on asthma in humans are conflicting.

In the present study, serum adiponectin levels of overweight/obese asthmatic children were found to be higher than in the other groups (Normal weight asthmatics and control). A significant positive correlation was found between BMI and serum adiponectin (*r* = 0.545, *p* =  < 0.001). This is consistent with Koksal et al. [30]. Meanwhile, in our study normal weight asthmatics had significantly lower adiponectin levels compared to control similar to previous researches [31-33]. Other studies, found normal levels of adiponectin in asthmatic children [34, 35].

Adiponectin was postulated to exert its action through either proinflammatory or anti-inflammatory mechanisms. It is possible that pro-inflammatory effects of adiponectin dominate under certain physiologic conditions while anti-inflammatory effects under others [36]. Increase in the function of adipose tissue in overweight/obese subjects leads to a systemic pro-inflammatory state in which serum concentrations of cytokines and soluble fractions in receptors and chemokines are increased. In patients with asthma, higher adiponectin levels create an inflammatory environment allowing for a stronger allergic response to antigens, resulting in worsening airway hyper-responsiveness and lower FEV_1_ [37]. It may in fact exacerbate the disease via allowing for naïve T-helper lymphocytes to differentiate into Th2 lymphocytes causing a more severe allergic response [37, 38]. In our study, a significant negative correlation between PEF and BMI was detected in obese/overweight asthmatics, also adiponectin level was inversely correlated to FEV1/FVC (although non-significant) but both finding could support the proinflammatory role of adiponectin in overweight/obese asthmatics. Same time, a higher adiponectin serum level in overweight/obese patients was accompanied by higher FEV1 compared to normal weight asthmatics. Adiponectin may be overexpressed in patients with bronchial inflammation to attenuate the inflammatory response through its anti-inflammatory effect [37] and thus, could have a protective role in asthmatic children [30]. It was shown that adiponectin infusion in mice can attenuate allergic airway inflammation and airway hyper-responsiveness by exerting anti-inflammatory effects [37]. The finding of better FEV_1_% and FVC % levels in the overweight/obese cases compared to normal weight asthmatics in our work with a positive correlation between each of FEV1% & FVC and BMI could support the anti-inflammatory role of adiponectin. Similar results reported by other studies [39, 40]; However, no differences in FEV_1_/FVC ratio were found between overweight/obese and normal weight asthmatic groups in our study that agreed with previous studies [41, 42].

Obesity is associated with airway dysanapsis; a condition characterized by unequal growth of lung parenchyma and airway caliber; that leads to larger lungs; with flows that are apparently normal but comparatively appear to be low [43]; due to reduced elasticity of the chest (mechanical obstruction) [44, 45]. Also, an association has been reported between excessive weight and total lung capacity (TLC) reduction [46], that was demonstrated in our study with negative correlation between PEF and BMI in overweight/obese cases.

Adiponectin has three different molecular weight isoforms: low (LMW), middle (MMW), and high (HMW). LMW exerts anti-inflammatory effects, while HMW activates pro-inflammatory pathways [47]. Adiponectin affects airway function through its receptors [48]. AdipoR_1_ is expressed in airway epithelial cells in chronic obstructive pulmonary disease [49]; while AdipoR_2_ is more highly expressed in people with asthma [48]. Allergen challenge decreased pulmonary expression of AdipoR_1_ and AdipoR_2_ and T-cadherin involved in transport of adiponectin into the lungs [50]. Abnormality in adiponectin receptors and lower expression of T-cadherin (a putative receptor of adiponectin) lead to abnormal adiponectin trafficking in the airway of overweight/obese asthmatic leading to better pulmonary functions FEV_1_ & FVC % observed in overweight/obese asthmatics compared to normal weight asthmatics [48]; as presented in our work.

Accordingly, our study observations could support the dual action postulation (pro- & anti-inflammatory) of adiponectin in overweight/obese asthmatics. The partial counteracting effects of both inflammations make it difficult to interpret this complex interplay between asthma and obesity.

According to roc curve result, with area under the curve 0.920, specificity = 82.8% and sensitivity = 82.6%, adiponectin can be considered a good predictor for obesity in asthmatic patients in our study, it can be used as a warning sign for asthmatics to watch their weight.

Levels of resistin have been reported to be either increased, unchanged, or decreased in murine and human obesity and type II diabetes. Only a few reports have investigated the effects of resistin in the modulation of inflammatory responses, showing that resistin upregulates expression of MCP-1, as well as vascular cell adhesion molecule 1 and ICAM-1, in endothelial cells [51]. In our study, no significant differences were found between resistin level and resistin/adiponectin ratio in normal weight asthmatics, overweight/obese asthmatics, and control group irrespective of sex; in agreement with previous researches [6, 52]. Some studies showed lower levels of resistin in atopic asthmatics than non-atopic asthmatics and controls [53]. Others reported higher resistin levels and higher resistin/adiponectin ratio in asthmatic subjects than in control subjects [54, 55].

It is obvious in our work that adiponectin is taking the upper hand in orchestrating asthma pathogenesis than resistin.

MCP-1 is thought to play an important role in the allergic inflammatory process, it may exert its proinflammatory effects through its C–c chemokine receptor type-2 (CCR2) receptors on antigen presenting cell (APC) and T-cells in lungs under influence of resistin and adiponectin [56]. Our results showed that MCP-1 levels were significantly lower in overweight/obese asthmatics compared to controls which is consistent with Keszei et al. [57], this might be attributed to increased use of oral steroids in overweight/obese asthmatics compared to normal weight (*p* = 0.003). On the contrary, previous research [58] demonstrated no statistically significant differences while others [59, 60] reported higher MCP-1 levels in asthmatic patients compared to controls. Reduction of MCP-1 level in serum was reported to be secondary to inhaled steroids and specific immunotherapy treatment in acute asthma [61, 62]. Some authors found reduction of its level in stable asthma and higher levels in acute asthma [63]. In our study, although 86.2% of normal weight asthmatics and 65.2% of overweight/obese asthmatics were on inhaled steroids, MCP-1 level did not show significant difference *p* = 0.318.

In our work, the mean age of asthma diagnosis (3.8 ± 2.9 years) preceded obesity (4.5 ± 2.4 years) suggestive of having TH2 phenotype in overweight/obese asthmatics. This is consistent with previous studies that reported development of obesity later in life of asthmatic individuals with early onset disease and higher IgE (high Th2 phenotype) [7, 48]. Increased IgE levels is usually associated with allergic symptoms [64]. It was significantly higher in normal weight and overweight/obese asthmatics compared to control; but no significant difference between both asthmatic groups, like previous study [65].

Our included asthmatic patients were mostly males. Studies found a higher frequency of asthma in males than females [66, 67]. Different patterns of lung function abnormalities in males and females existed due to different pattern of lung growth and fat deposition [7]. Males compared to females are known to have reduced airway caliber relative to the lung size early in life, and likely contributes to male infants having greater early wheezing and airway hyperresponsiveness [68]. Obesity is a trigger factor for asthma in rural areas as low socioeconomic standard is a risk factor for overweight and obesity [69].

Even though our data agreed with a previous study [70] that increased BMI had little effect on symptom control, degree of asthma severity and exacerbation risk in overweight/obese asthmatic children; this differs from what other studies published [71].

### Limitations to our study

Small sample size and the lack of overweight/obese control group which may offer better statistical results.

## Conclusion

This work could suggest that adiponectin may play a role in overweight/obese asthma phenotype where it is possible to have a dual action (pro & anti- inflammatory). It seems that resistin had no role in asthma pathogenesis. Further studies are needed to prove/disprove this observation.

## Recommendations

- Studying these hormones in both serum and bronchial lavage for better understanding of adipokines target cells and signaling pathways specially adiponectin molecular forms and receptors. Follow up of normal weight asthmatics by serum adiponectin for early prediction of obesity.

## Data Availability

The datasets generated and/or analyzed during the current study are not publicly available due to confidentiality on handling the database and guaranteeing the privacy of participants according to the ethical committee but are available from the corresponding author on reasonable request.

## Abbreviations

APC: Antigen presenting cell
ATS: American thoracic society
ATS/ERS: American thoracic society/european respiratory society
BMI: Body mass index
CCR2: C–c chemokine receptor type-2
ELISA: Enzyme-linked immunosorbent assay
FEF25-75%: Forced expiratory flow at middle half of fvc
FEV1: Forced expiratory volume in 1 s
FVC: Forced vital capacity
GINA: Global initiative for asthma
ICS: Inhaled corticosteroids
IgE: Immunoglobulin E
MCP-1: Monocytes chemoattractant protein 1
NF-κβ: Nuclear factor kappa-light-chain-enhancer of activated B cells)
NRC: National research centre
PEF: Peak expiratory flow
PFT: Pulmonary function test
Th: T-helper
ROC: Receiver operating characteristic analysis
SPSS: Statistical Package for social sciences
